# Predictive value of nutritional indices for left atrial thrombus in patients with valvular atrial fibrillation

**DOI:** 10.1186/s12872-023-03493-4

**Published:** 2023-10-27

**Authors:** You Zhou, Erpeng Liang, Jifang Ma, Xianqing Wang, Haixia Fu

**Affiliations:** Heart center of Henan Provincial People’s Hospital, Central China Fuwai Hospital, Central China Fuwai Hospital of Zhengzhou University, NO.1 Fuwai Avenue, Zhengdong New District, Zhengzhou, 450003 Henan China

**Keywords:** Nutritional indices, Left atrial thrombus, Valvular atrial fibrillation, Inflammation

## Abstract

**Background:**

The prognostic nutritional index (PNI) and geriatric nutritional risk index (GNRI) are well known indicators for adverse outcomes in various diseases, but there is no evidence on their association with the risk of left atrial thrombus (LAT) in patients with valvular atrial fibrillation (VAF).

**Methods:**

A comparative cross-sectional analytical study was conducted on 433 VAF patients. Demographics, clinical characteristics and echocardiographic data were collected and analyzed. Patients were grouped by the presence of LAT detected by transesophageal echocardiography.

**Results:**

LAT were identified in 142 patients (32.79%). The restricted cubic splines showed an L-shaped relationship between PNI and LAT. The dose-response curve flattened out near the horizontal line with OR = 1 at the level of 49.63, indicating the risk of LAT did not decrease if PNI was greater than 49.63. GNRI was negative with the risk of LAT and tended to be protective when greater than 106.78. The best cut-off values of PNI and GNRI calculated by receiver operating characteristics curve to predict LAT were 46.4 (area under these curve [AUC]: 0.600, 95% confidence interval [CI]:0.541–0.658, P = 0.001) and 105.7 (AUC: 0.629, 95% CI:0.574–0.684, P<0.001), respectively. Multivariable logistic regression analysis showed that PNI ≤ 46.4 (odds ratio: 2.457, 95% CI:1.333–4.526, P = 0.004) and GNRI ≤ 105.7 (odds ratio: 2.113, 95% CI:1.076–4.149, P = 0.030) were independent predictors of LAT, respectively.

**Conclusions:**

Lower nutritional indices (GNRI and PNI) were associated with increased risk for LAT in patients with VAF.

## Introduction

Atrial fibrillation (AF) is the most common cardiac arrhythmia with increasing health-related burden. The current prevalence of AF is between 2% and 4%, which is expected to continue rising owing to extended longevity [1]. AF is associated with an increased risk of stroke and thromboembolism, resulting to substantial morbidity and mortality. Among different underlying diseases, AF patients with valvular heart disease are at the highest risk of ischemic stroke [2, 3]. Valvular atrial fibrillation (VAF) predominantly affects large populations in many low- and middle- income countries. In a global registry study, VAF accounted for 8.8% of AF patients in Western Europe, but 32.6% in Africa and 46.7% in India [4]. To make matters worse, stroke related to VAF occurs in a much younger population, leading to consequent loss of manpower and resultant heavy economic burdens. Disorganized electrical activity in the atrium is the main characteristic of AF, which prompts ineffective contraction and stasis of blood. Blood stasis gives rise to thrombogenic tendency in the left atrium. It has been well recognized that the left atrial thrombus (LAT) formation is the primary cause of stroke in patients with AF [5]. Therefore, it is essential to identify VAF patients at high risk of LAT and provide appropriate treatment to protect them from stroke.

Malnutrition is a common status in patients with chronic diseases, especially in low- and middle- income countries. More than a third of AF patients have a moderate to high risk of malnutrition [6]. Nutritional deficiencies may also be a driver of disease progression as part of a vicious cycle associated with chronic systematic inflammation, neurohumoral activation and cachexia [7]. Various systems and scores have been proposed for evaluation of malnutrition. Among theses assessment tools, the prognostic nutritional index (PNI) and geriatric nutritional risk index (GNRI) have been widely used for screening malnutrition in clinical practice. Malnutrition determined by these scoring methods is an independent predictor of increased mortality in AF patients [6, 8]. However, the predictive value of PNI and GNRI for LAT in patients with VAF remains unknown.

Accordingly, we conducted this study to investigate the association between nutritional indices and LAT in patients with VAF undergoing transesophageal echocardiography (TEE).

## Methods

### Study population

**Fig. 1:**
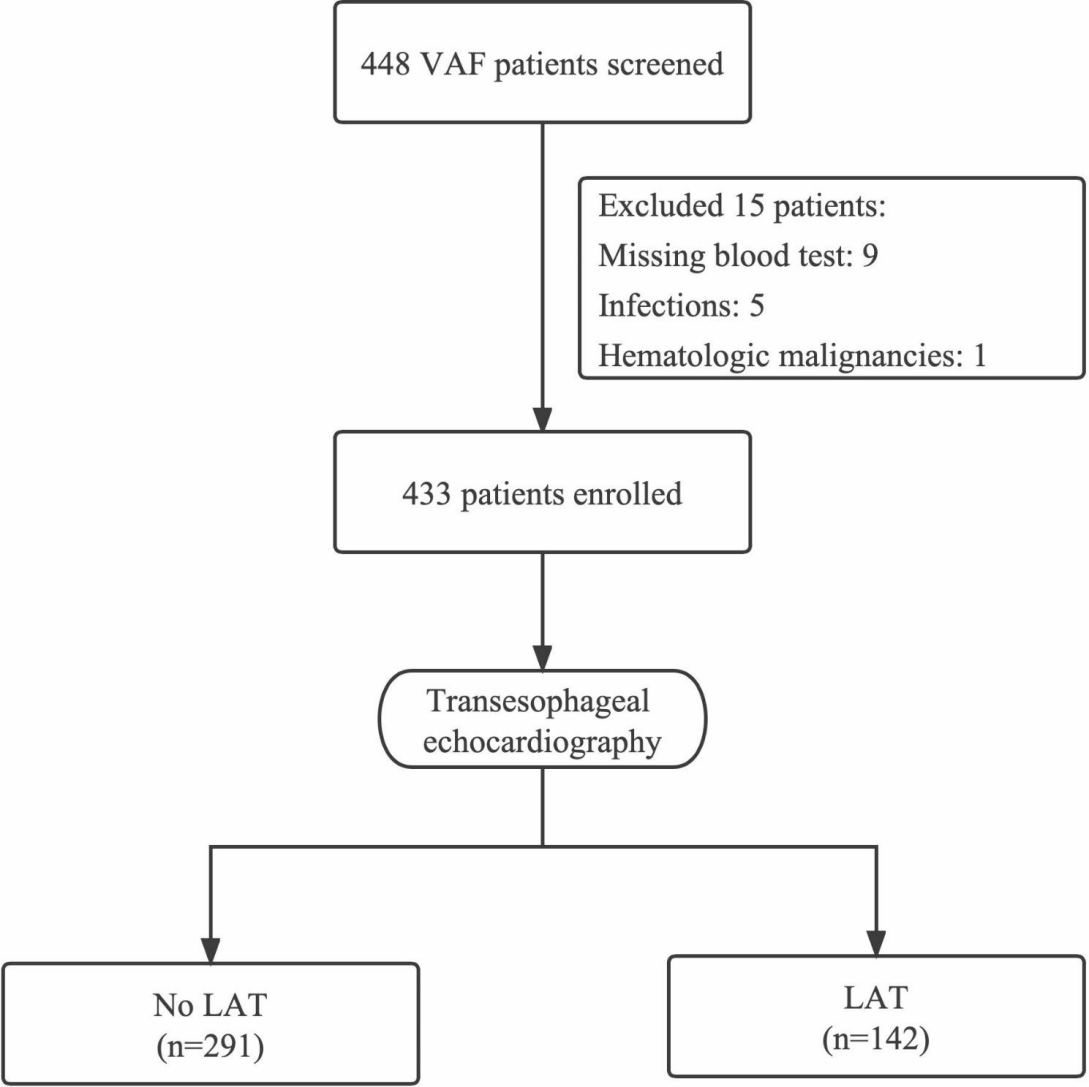
Flow diagram showing screening and recruitment of the study population. VAF, valvular atrial fibrillation; LAT, left atrial thrombus

Consecutive hospitalized patients with VAF who undertook TEE to determine LAT in Henan Provincial People’s Hospital between January 2015 and September 2022 were enrolled in our study. VAF is defined according to guideline as AF patients with moderate or severe mitral stenosis and those with mechanical prosthetic heart valve(s) [1]. First-diagnosed AF was defined as AF not diagnosed previously, irrespective of its duration or the presence/severity of AF-related symptoms [1]. Patients with missing blood test, acute and/or chronic infection and hematologic malignancies were excluded. The flowchart of patient inclusion was shown in Fig. 1. The study protocol was approved by the Ethics Committee of the Henan Provincial People’s Hospital. The need for informed consent was waived by the Ethics Committee of the Henan Provincial People’s Hospital, because of the retrospective nature of the study.

### Data collection

Patients’ clinical data prior to TEE were retrieved from medical records, including age, gender, type of atrial fibrillation, comorbidities (coronary heart disease, heart failure, hypertension, diabetes, history of stroke), oral anticoagulant therapy, laboratory variables and echocardiographic parameters. Body mass index (BMI) was calculated as weight divided by height squared (kg/m^2^). Venous blood samples after 12 h fasting were collected from each patient after admission and before TEE examination. All biochemical tests were measured in the laboratory of the testing center for examination by standard techniques. Estimate glomerular filtration rate was calculated using the Cockcroft-Gault equation.

### Nutritional indices equations

Nutritional indices were calculated using these equations:

PNI = 10 × serum albumin (g/dl) + 0.005 × total lymphocyte count (mm^3^) [9].

GNRI = 14.89 × serum albumin (g/dl) +41.7 × (body weight/ideal body weight) [10].

### Echocardiographic parameters

After admission, transthoracic echocardiography and TEE were performed according to international guidelines. LAT referred to a highly reflective and well-circumscribed mass with uniform consistency and different texture from the atrial wall in TEE [11]. The peak and mean gradients, which were measured by the mitral inflow velocities with continuous wave Doppler ultrasound scanning from the apical view, were used to evaluate the severity of mitral stenosis. The area of mitral valve was calculated in the short-axis view by pressure half-time method and planimetry of the mitral valve orifice in early diastole phase. Moderate or severe mitral stenosis referred to patients with a valve area of < 1.5 cm^2^ or a mean gradient of > 5 mmHg. Left atrial diameter (LAD) and left ventricular ejection fraction were measured from M-mode or 2D view in the parasternal long-axis projection.

### Statistical analysis

Normally distributed continuous variables were presented as mean (± standard deviation) and compared by Student’s t tests. Continuous variables with non-normally distribution were represented as median (inter-quartile range), and compared by the Mann-Whitney U test. Categorical variables in each group were presented as numbers and percentages, and compared by the Chi-square test. Spearman test was utilized for describing the correlation between nutritional indices and clinical variables. Restricted cubic splines were plotted to illustrate the dose-response association between nutritional indices and the risk of LAT. Four knots were placed at the 5th, 35th, 65th, and 95th percentiles of nutritional indices. Receiver operating characteristic (ROC) curves were established to evaluate predictive ability of nutritional indices on risk of LAT and identify the optimal cut-off values by the highest Youden index. Area under the curves (AUC) were calculated and compared by DeLong test. Multiple logistic regression analyses were performed with an enter regression model in which nutritional indices and each variable with a P value < 0.05 (based on the univariable analysis) were entered. Odds ratio (OR) and 95% confidence interval (CI) were calculated to demonstrate the risk of LAT. All significance tests were two-tailed and p values < 0.05 were considered statistically significant. SPSS Statistics 26.0 (SPSS, Chicago, IL, USA) and R 4.1.2 (R Core Team, Vienna, Austria) were used to perform the statistical analysis.

## Results

### Patient characteristics

**Fig. 2:**
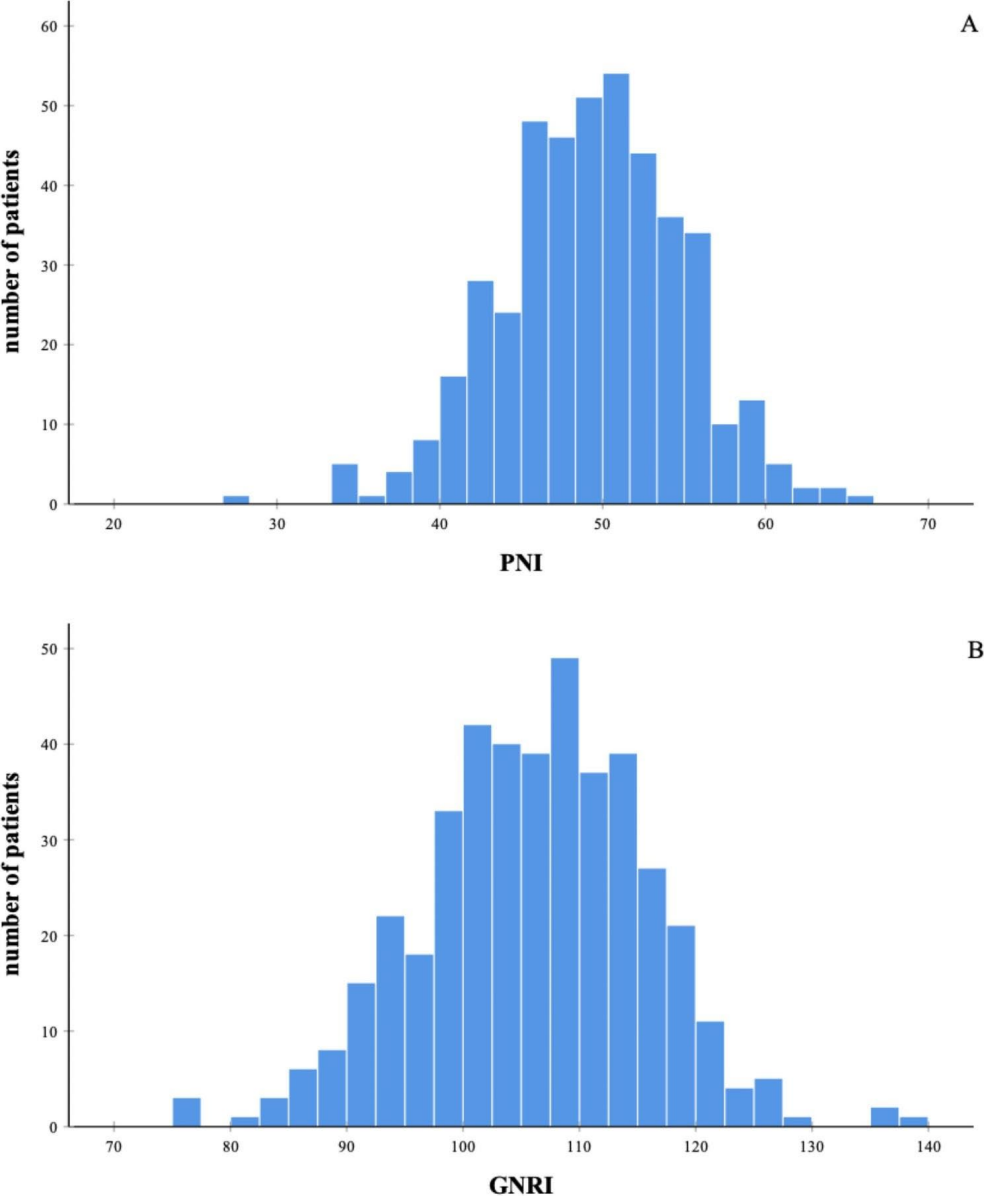
Distributions of nutritional indices among patients with valvular atrial fibrillation. (A) Distribution of the prognostic nutritional index (PNI). (B) Distribution of the geriatric nutritional risk index (GNRI).

A total of 433 patients were enrolled in this study. The average age of included patients was 56.86 ± 9.13 years, and only 36.5% were men. 32.8% of these included patients received oral anticoagulant at admission. All anticoagulated participants received warfarin. LAT were found in 142 patients (32.79%). Patients with LAT were more likely to be male, had diabetes and heart failure, with larger LAD, lower BMI and higher level of C-reactive protein (CRP), than those without LAT (Table 1). The average PNI score and GNRI score were 49.34 ± 5.61 and 106.08 ± 9.67, respectively. Both PNI and GNRI were significantly lower in patients with LAT. The distributions of the nutritional indices were shown in Fig. 2.

**Table 1:** Baseline characteristics of the study population stratified by left atrial thrombus

	Total (n = 433)	No LAT (n = 291)	LAT (n = 142)	P-value
Male, n (%)	158(36.5%)	96(33.0%)	62(43.7%)	0.030
Age (years)	56.86 ± 9.13	57.08 ± 8.97	56.41 ± 9.47	0.474
Body mass index(Kg/m^2^)	23.83 ± 3.55	24.13 ± 3.64	23.24 ± 3.29	0.015
Paroxysmal atrial fibrillation, n (%)	44(10.2%)	34(11.7%)	10(7.0%)	0.133
First-diagnosed atrial fibrillation, n (%)	261(60.3%)	168(57.7%)	93(65.5%)	0.121
Moderate/severe mitral stenosis, n (%)	419(96.8%)	279(95.9%)	140(98.6%)	0.134
Coronary heart diseases, n (%)	20(4.6%)	14(4.8%)	6(4.2%)	0.785
Heart failure, n (%)	287(66.3%)	183(62.9%)	104(73.2%)	0.032
Hypertension, n (%)	62(14.3%)	45(15.5%)	17(12.0%)	0.330
Diabetes, n (%)	28(6.5%)	13(4.5%)	15(10.6%)	0.015
Stroke, n (%)	80(18.5%)	48(16.5%)	32(22.5%)	0.128
Oral anticoagulant, n (%)	142(32.8%)	101(34.7%)	41(28.9%)	0.225
Left atrium diameter (mm)	55.04 ± 11.37	54.25 ± 11.17	56.66 ± 11.63	0.045
LVEF (%)	56.79 ± 8.04	57.33 ± 8.07	55.72 ± 7.92	0.072
eGFR( mL/min/1.73 m^2^)	88.61 ± 17.84	89.08 ± 17.12	87.67 ± 19.27	0.441
Albumin (g/L)	40.87 ± 4.26	41.38 ± 4.00	39.84 ± 4.58	0.001
Triglyceride (mmol·L^− 1^)	1.35 ± 0.70	1.40 ± 0.72	1.25 ± 0.64	0.058
Total cholesterol (mmol/L)	4.00 ± 1.00	4.04 ± 1.02	3.92 ± 0.96	0.301
HDL-C (mmol/L)	1.11 ± 0.31	1.13 ± 0.29	1.06 ± 0.33	0.061
LDL-C (mmol/L)	2.54 ± 1.61	3.58 ± 1.90	2.47 ± 0.80	0.539
Fasting blood glucose (mmol/L)	4.85 ± 1.33	4.81 ± 1.24	4.93 ± 1.50	0.395
C-reactive protein (mg/L)	1.76(0.65,5.20 )	1.16(0.53,2.94 )	3.74(1.39,7.67 )	< 0.001
PNI	49.34 ± 5.61	50.03 ± 5.17	47.93 ± 6.20	0.001
GNRI	106.08 ± 9.67	107.41 ± 9.70	103.39 ± 9.05	< 0.001

Abbreviations: LAT, left atrial thrombus; LVEF, Left ventricular ejection fraction; eGFR, estimated glomerular filtration rate; HDL-C, High density lipoprotein cholesterol; LDL-C, Low density lipoprotein cholesterol; PNI, Prognostic nutrition index; GNRI, Geriatric nutritional risk index.

### The correlation between nutritional indices and baseline variables

In correlation analysis, PNI and GNRI were positively correlated with BMI (r = 0.142, P = 0.003; r = 0.753, P < 0.001, respectively), triglyceride (r = 0.261, P < 0.001; r = 0.291, P < 0.001, respectively), low density lipoprotein cholesterol (r = 0.213, P < 0.001; r = 0.107, P = 0.040, respectively), fasting blood glucose (r = 0.129, P = 0.011; r = 0.257, P < 0.001, respectively) and negatively with CRP (r=-0.294, P < 0.001; r=-0.150, P = 0.009, respectively) (Table 2). Only PNI was positively correlated with high density lipoprotein cholesterol (r = 0.143, P = 0.005).

**Table 2:** Correlation analysis between prognostic nutrition index and baseline variables

	Prognostic nutrition index		Geriatric nutritional risk index	
	Correlation Coefficient	P-value	Correlation Coefficient	P-value
Age	-0.062	0.199	0.004	0.931
Body mass index	0.142	0.003	0.753	<0.001
Left atrium diameter	-0.088	0.079	-0.046	0.365
Left ventricular ejection fraction	0.081	0.125	0.057	0.287
Estimated glomerular filtration rate	-0.040	0.408	-0.048	0.317
Triglyceride	0.261	<0.001	0.291	<0.001
Total cholesterol	0.248	<0.001	0.140	0.007
High density lipoprotein cholesterol	0.143	0.005	-0.001	0.980
Low density lipoprotein cholesterol	0.213	<0.001	0.107	0.040
Fasting blood glucose	0.129	0.011	0.257	<0.001
C-reactive protein	-0.294	<0.001	-0.150	0.009

### The dose-response association between nutritional indices and the risk of LAT

**Fig. 3:**
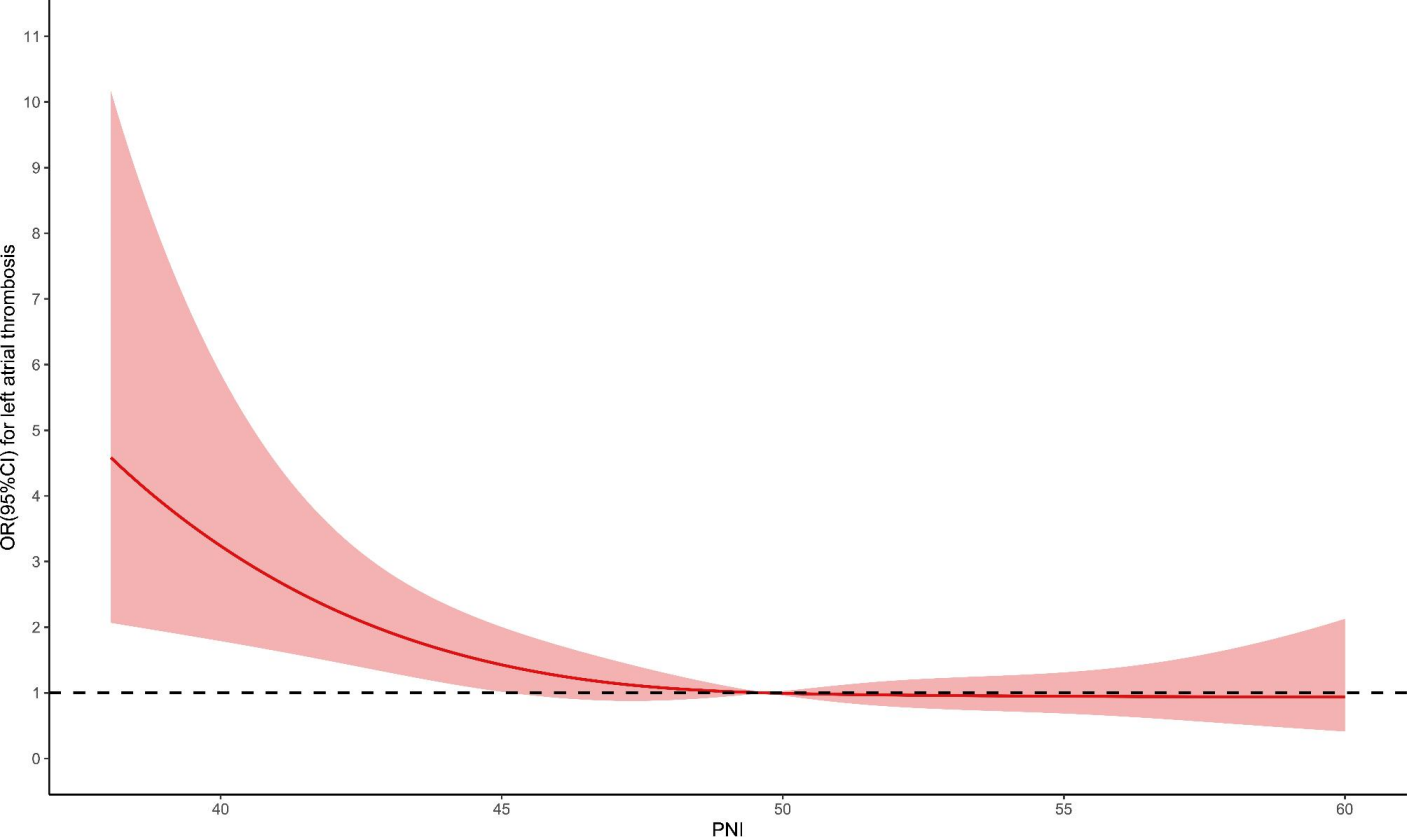
Dose-response curve of PNI and the risk of left atrial thrombus. PNI, prognostic nutritional index; OR, odds ratio; CI, confidence interval

**Fig. 4:**
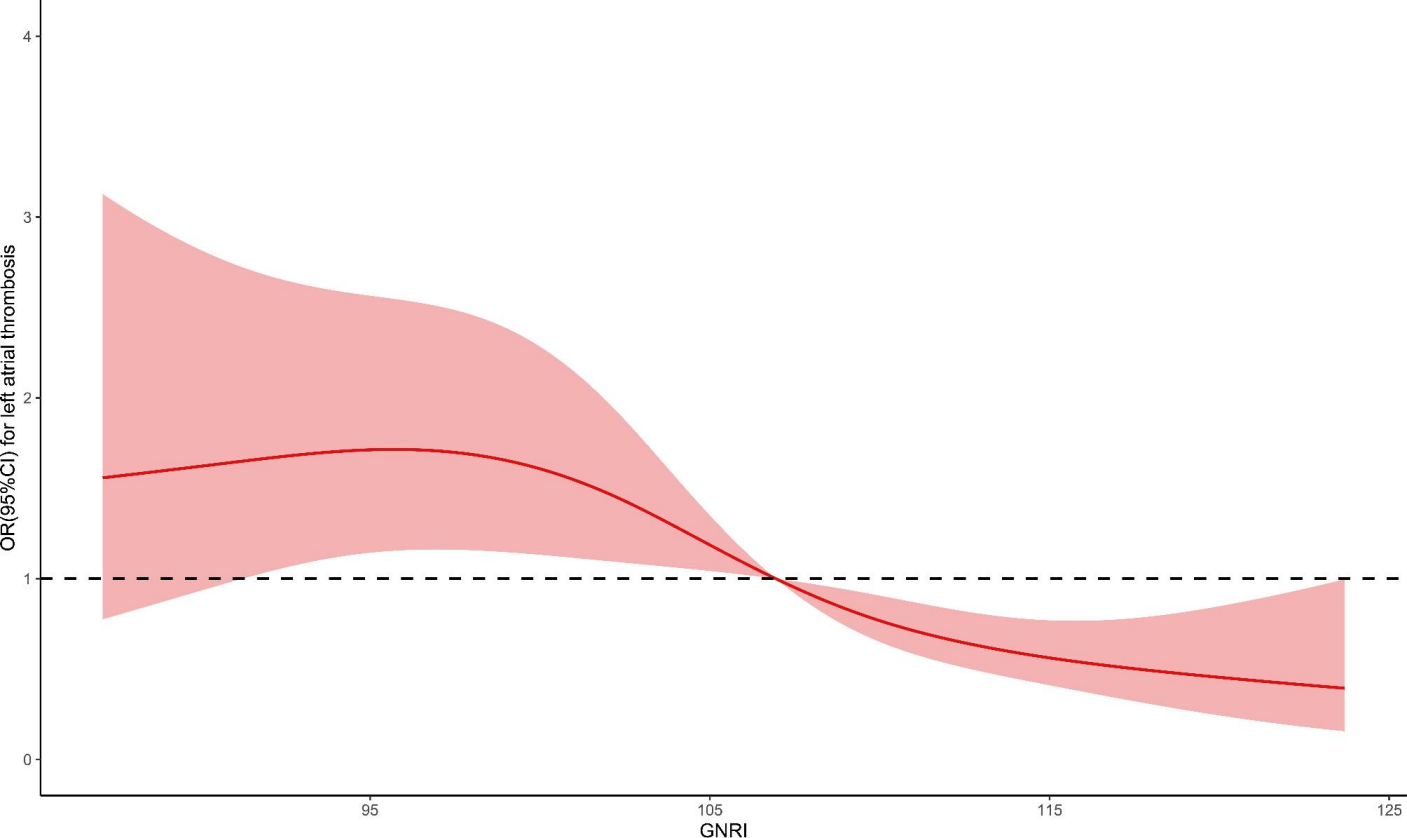
Dose-response curve of GNRI and the risk of left atrial thrombus. GNRI, geriatric nutritional risk index; OR, odds ratio; CI, confidence interval

Dose-response curves were constructed to continuously assess the association of nutritional indices with risk of LAT. There existed a non-linear and L-shaped relationship between PNI and the risk of LAT (P for non-linearity < 0.001). With the increase of PNI, the OR decreased until it reached 49.63; thereafter, the OR tended to the horizontal line with OR = 1 (Fig. 3). GNRI had a non-linear correlation and was negative with the risk of LAT (P for non-linearity < 0.001). The OR decreased until it reached 106.78 ; thereafter, higher GNRI gradually showed a protective role (Fig. 4).

### Predictive value of the Nutritional Indices for LAT

ROC analysis revealed that the optimal cutoff values of PNI and GNRI for predicting LAT were 46.4 (AUC: 0.600, 95%CI:0.541–0.658, P = 0.001; sensitivity 43.0%, specificity 76.6%) and 105.7 (AUC: 0.629, 95%CI:0.574–0.684, P < 0.001; sensitivity 61.0%, specificity 61.2%), respectively. According to pair-wise comparison of the AUC, PNI and GNRI had the similar predictive value (P = 0.286). The univariate logistic regression analysis demonstrated that male, BMI, heart failure, diabetes, larger LAD, higher serum levels of CRP, PNI < 46.4 and GNRI < 105.7 were significantly associated with higher incidence of LAT, while higher PNI and GNRI were associated with lower risk (Table 3). After adjusting gender, BMI, heart failure, diabetes, LAD, and CRP, PNI < 46.4 (OR: 2.457, 95% CI:1.333–4.526, P = 0.004) and GNRI < 105.7 (OR: 2.113, 95% CI:1.076–4.149, P = 0.030) remained independent predictors of LAT. As continuous variates, higher PNI (OR: 0.919, 95% CI:0.869–0.971, P = 0.003) and higher GNRI (OR: 0.935, 95% CI:0.891–0.980, P = 0.005) were significantly related with lower risk of LAT after adjusting for the same factors (Table 3).

**Table 3:** Logistics regression analysis to identify the in predictors of left atrial thrombus

	OR (95% CI)	P-value
Univariate analysis		
Male	1.574(1.043–2.377)	0.031
Age	0.992(0.970–1.014)	0.473
Body mass index	0.929(0.876–0.986)	0.015
Paroxysmal atrial fibrillation	1.746(0.837–3.644)	0.137
Coronary heart diseases	0.873(0.328–2.321)	0.785
Heart failure	1.615(1.039–2.511)	0.033
Hypertension	0.743(0.409–1.352)	0.331
Diabetes	2.526(1.167–5.465)	0.019
Stroke	1.473(0.893–2.430)	0.130
Oral anticoagulant	0.764(0.494–1.181)	0.225
Left atrium diameter	1.018(1.000-1.037)	0.049
LVEF	0.976(0.950–1.002)	0.074
eGFR	0.996(0.985–1.007)	0.440
Triglyceride	0.725(0.518–1.014)	0.060
Total cholesterol	0.892(0.717–1.108)	0.301
HDL-C	0.492(0.233–1.038)	0.062
LDL-C	0.949(0.798–1.128)	0.552
Fasting blood glucose	1.069(0.916–1.248)	0.395
C-reactive protein	1.064(1.028–1.101)	<0.001
PNI	0.934(0.899–0.969)	<0.001
PNI < 46.4	2.470(1.608–3.793)	<0.001
GNRI	0.956(0.935–0.978)	<0.001
GNRI < 105.7	2.421(1.603–3.656)	<0.001
Multivariate analysis		
PNI^a^	0.919(0.869–0.971)	0.003
PNI < 46.4^a^	2.457(1.333–4.526)	0.004
GNRI^a^	0.935(0.891–0.980)	0.005
GNRI < 105.7^a^	2.113(1.076–4.149)	0.030

Abbreviations: OR, odds ratio; CI, confidence interval; LVEF, Left ventricular ejection fraction; eGFR, estimated glomerular filtration rate; HDL-C, High density lipoprotein cholesterol; LDL-C, Low density lipoprotein cholesterol; PNI, Prognostic nutrition index; GNRI, Geriatric nutritional risk index.

a: Adjusted for gender, body mass index, Heart failure, Diabetes, Left atrium diameter, C-reactive protein.

## Discussion

This study is the first to explore the predictive value of nutritional indices for LAT in patients with VAF. Our principal finding was that lower nutritional indices were associated with increased risk of LAT. In addition, the two indices had different dose-response association with the occurrence of LAT. We found an L-shaped relationship between PNI and LAT. The dose-response curve flattened out near the horizontal line with OR = 1 at 49.63, indicating the risk of LAT did not decrease if PNI was greater than 49.63. GNRI was negative with the risk of LAT in a non-linear form and tended to be protective when GNRI was greater than 106.78.

To date, there existed limited information about the association between nutritional status and stroke or LAT in patients with AF. Most of these studies focused on the ‘obesity paradox’ in patients with non-valvular AF (NVAF), which referred that obesity might be associated with a lower thromboembolic risk [12–14]. Only one study investigated the relationship between nutritional indices and LAT in patients with AF [14]. In Wang and his colleagues’ study including 406 patients with NVAF, PNI was an independent predictor for LAT (OR: 0.89, P = 0.007), the optimal cutoff value of the PNI for predicting LAT was 48.0 (AUC: 0.68,P < 0.001) and the risk of LAT in patients with PNI ≤ 48.0 was 2.57-fold higher than that in those with PNI > 48.0 [15]. Similar to this study, our research confirmed the predictive value of PNI for LAT formation in a VAF population. Furthermore, we also found that GNRI was also an independent risk factor for LAT.

PNI and GNRI are more than nutritional indicators. Evidence showed serum albumin regulated the body’s inflammatory response and had antioxidant properties through multiple binding sites and its ability to trap free oxygen radicals [16, 17]. Previous investigations found that level of serum albumin was negatively correlated with serum inflammatory biomarkers [18]. Hypoalbuminemia was also found to increase susceptibility to thrombotic events in patients with cardiovascular disorders [19]. Low lymphocyte count is common in cardiovascular diseases as a chronic inflammatory response. Several mechanisms for this phenomenon have been postulated, the most important being: (1) redistribution of lymphocytes from peripheral blood to other sites (infiltration of activated T-cells in heart tissues) and (2) accelerating cell death due to apoptosis [20]. Low lymphocyte count is also associated with poor prognosis in patients with a wide variety of diseases [20]. Our study and previous researches found that PNI and GNRI correlated with inflammatory markers such as CRP or neutrophil-to-lymphocyte ratio [15, 21]. The above evidence suggested that PNI and GNRI could not only evaluate the nutritional status but also effectively reflect the immune and inflammation status of the body. The presence of systemic inflammation in VAF patients was found by measuring serum inflammatory biomarkers or histopathological examination of the left atrium [22, 23]. Inflammation also caused and exaggerated the structural and electrical remodeling of the atria, leading to ineffective irregular contraction and blood stasis [22, 23]. Blood stasis in the left atrium resulted in thrombogenic tendency in patients with VAF. A recent study found higher level of serum inflammatory biomarkers to be statistically significant predictors of LAT in patients with VAF [24]. Therefore, in view of our study and findings from previous investigations, systemic inflammation might also contribute to the association between the indices examined in this study and LAT in VAF patients. However, the major difference between the two indices is that GNRI includes anthropometric factors (the ratio of body weight to ideal body weight). GNRI is a multidimensional indicator. Besides systemic inflammation, metabolic factors also have large impact on this score. PNI only consists of serum markers, so it is more susceptible to systemic inflammation. We postulated that lower level of systemic inflammation reduced the risk of LAT, but it could not be protective. This might contribute to the L-shaped dose-response curve between PNI and LAT. The different dose-response curves of the two indices indicated that, in patients with over-nutrition status such as overweight, PNI might not discriminate those with high risk of LAT while GNRI could. Therefore, this might be an advantage of GNRI over PNI.

Evaluation for nutrition status in patients with VAF and performing early interventions for malnutrition might be of high importance for their prognosis. Malnutrition is not only a marker of disease severity but also a contributor of disease progression. Long-term chronic disease, such as VAF, leads to malnutrition, which expedites the progression of the disease [7]. Sunaga et al. found improvement in nutritional status during hospitalization were associated with better prognosis in patients with malnutrition and heart failure [25]. Felipe et al. showed polyunsaturated fatty acids and antioxidant vitamins could reduce oxidative and nitrosative stress and prevent Cx40/Cx43 lateralization in atrial tissue to avoid AF occurrence [26]. Clinical trial had also demonstrated that micronutrient supplementation might prevent ischaemic events in patients with high risk of stroke [27]. Similar effects may be achieved to reduce thromboembolic risk when nutritional treatment is administered to patients with VAF. Further studies are needed to determine whether nutrition interventions can reduce thromboembolic risk in patients with VAF.

The prevalence of LAT varied in prior reports due to the proportion of receiving anticoagulants. Left atrial thrombus were found in approximately 5–27% of AF patients without anticoagulation therapy [28]. In our study, the occurrence of LAT was 32.79% with a relative low rate of oral anticoagulation therapy(32.8%). 60.3% patients in the present study were diagnosed AF for the first time. This partly contribute to the low prevalence of anticoagulation. Underuse of oral anticoagulation in VAF was also observed in other researches [28, 29]. In nationwide Danish registries from 2000 to 2018, only 54% VAF patients received oral anticoagulant therapy [29]. VAF patients had more commodities, such as heart failure and malnutrion, increasing the bleeding risk. It might cause the hesitation to anticoagulant therapy.

All VAF patients are indicated for anticoagulation therapy. Warfarin have been proven to be effective in the prevention of LAT [1]. However, in patients with nutritional problems, drug metabolism and food interaction may influence the efficacy of warfarin for thromboembolic prevention. In our study, participants with lower nutritional indices had higher risk of LAT, but those patients were also considered with higher risk of bleeding in previous research [30]. Therefore, patients with lower PNI or GNRI required more frequent follow-up and close monitoring of time in therapeutic range. Measuring those nutritional indices may help anticoagulant strategy in clinical practice.

Despite the crucial findings being mentioned, our study had some limitations. First, it was a retrospective observational study including a small number of hospitalized patients. The enrollment of patients was not randomized, there might exist selection bias. The malnutrition status of hospitalized patients with VAF did not precisely reflect the malnutrition status in the overall population with VAF. More prospective studies with larger samples are needed to further validate our findings. Secondly, besides LAD, other echocardiographic paramters for thromboembolic risk in AF patients, such as left atrial volume index, left atrial appendage area and left atrial appendage wall motion velocity, were not routinely measured in our center [31]. In addition, in the anticoagulated patients enrolled in our study, we did not have information about the quality of anticoagulant control (e.g.time in therapeutic range for patients receiving warfarin and actual intake of oral anticoagulants), which impact the risk of LAT. Furthermore, we only used two simple indices to evaluate the nutritional status but did not compare the predictive value of more comprehensive and complex nutritional screening tools [32]. Finally, the nutritional indices were assessed only at admission; subsequent temporal changes and their relationship with endpoints could not be assessed. Dynamic monitoring of these indices could provide more information.

## Conclusions

In summary, we reported that lower nutritional indices (GNRI and PNI) were associated with higher risk for LAT in a VAF population. These two indices could be used as cost-effective predictors for thromboembolic risk in patients with VAF due to their low cost and easy accessibility, especially in low- and middle- income countries.

## Data Availability

The datasets generated and analysed during the current study are not publicly available due to the Henan Provincial People’s Hospital regulations, but are available from the corresponding author on reasonable request.

## List of abbreviations

AF: Atrial fibrillation
NVAF: Non-valvular atrial fibrillation
VAF: Valvular atrial fibrillation
LAT: Left atrial thrombus
CRP: C-reactive protein
BMI: Body mass index
PNI: Prognostic nutritional index
GNRI: Geriatric nutritional risk index
TEE: Transesophageal echocardiography
LAD: Left atrium diameter
LVEF: Left ventricular ejection fraction
ROC: Receiver operating characteristic curve
AUC: Area under the curve
OR: Odds ratio
CI: Confidence Interval
